# 

**Published:** 1883-10

**Authors:** 


### CONTENTS.

| | Page. | | Page. |
|---|---|---|---|
| To Our Readers | 205 | Stammering | 215 |
| Fat and Lean | 207 | Simple Remedies | 216 |
| Fever and Ague | 208 | Dangers of Sea Bathing | 218 |
| The Hot Water Cure | 209 | Food Adulteration | 220 |
| Insidious Destroyers | 211 | When to Take Medicine | 221 |
| Cancer | 213 | Henry Ward Beecher's Sermons | 222 |
| Bronchitis | 213 | | |

